# Bilateral Macular Roth Spots as a Manifestation of Subacute Endocarditis

**DOI:** 10.1155/2015/493947

**Published:** 2015-12-29

**Authors:** Karolina Ceglowska, Katarzyna Nowomiejska, Agnieszka Kiszka, Michael J. Koss, Ryszard Maciejewski, Robert Rejdak

**Affiliations:** ^1^Department of General Ophthalmology, Medical University, 20-079 Lublin, Poland; ^2^Department of Ophthalmology, University of Heidelberg, 69120 Heidelberg, Germany; ^3^Human Anatomy Department, Medical University, 20-090 Lublin, Poland; ^4^Department of Experimental Pharmacology, Medical Research Centre, Polish Academy of Sciences, 02-069 Warsaw, Poland

## Abstract

A 42-year-old man presented with a 2-day history of impaired vision in the right eye (OD). The best corrected visual acuity (BCVA) (LogMAR) was 1.1 for the right eye and 0.0 for the left eye (OS). Fundus examination revealed white-centered hemorrhages resembling Roth spots in both macular regions. The spectral-domain optical coherence tomography (SD-OCT) showed intraretinal pseudocysts and hyperreflective deposits in the areas corresponding to the Roth spots. Conducted blood tests revealed elevated D-dimer concentration, increased total number of neutrophils, high C-reactive protein concentration, and elevated erythrocyte sedimentation rate. Procalcitonin concentration, platelet count, and body temperature were within normal ranges. A blood culture was ordered and yielded* Streptococcus mitis* and intravenous antibiotics were started immediately. The patient started complaining of chest and left calf pain. The systemic examination revealed infective endocarditis accompanied by bicuspid aortic valve and paravalvular abscess formation. The patient underwent cardiac surgery with mechanical aortic valve implantation. After recovery, the patient's visual acuities improved fully. Control ophthalmic examination, including SD-OCT, showed no abnormalities.

## 1. Introduction

White-centered retinal hemorrhages, also known as Roth spots, are a nonspecific retinal finding and may be an ocular manifestation of many systemic disorders including those that may be life-threatening. It is believed that these white-centered hemorrhages are the result of retinal capillary ruptures and intraretinal blood leakage. The white lesions were proven histopathologically to be the platelet-fibrin thrombi, an indication of the reparative process of the capillary rupture [1]. Therefore, conditions that may lead to Roth spots are those that cause increased capillary fragility or intravascular coagulopathy, such as bacterial endocarditis, leukemia, or anemia.

## 2. Case Presentation

**Figure 1:**
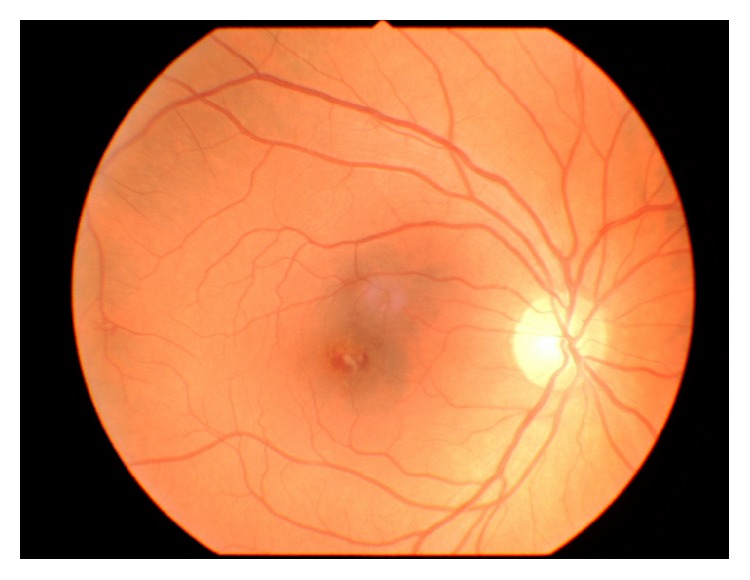
Color fundus photograph of the right eye showing central macular Roth spot.

**Figure 2:**
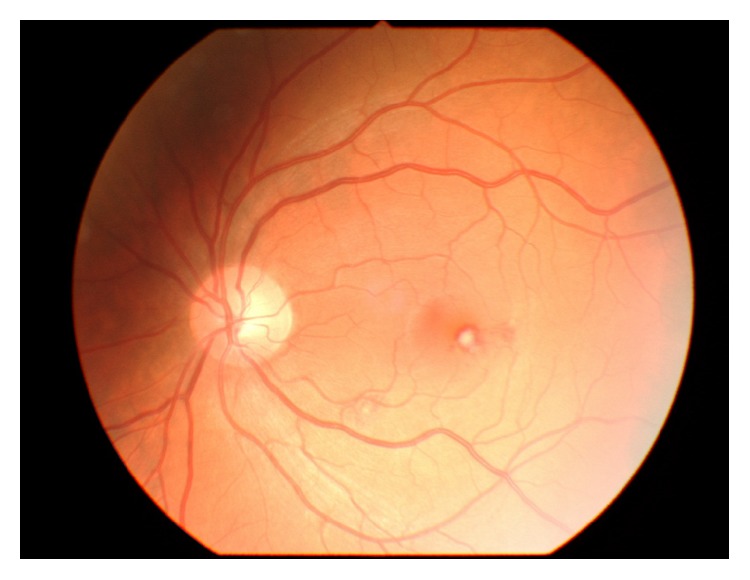
Color fundus photograph of the left eye presenting Roth spots in the macula and the inferior temporal branches region.

**Figure 3:**
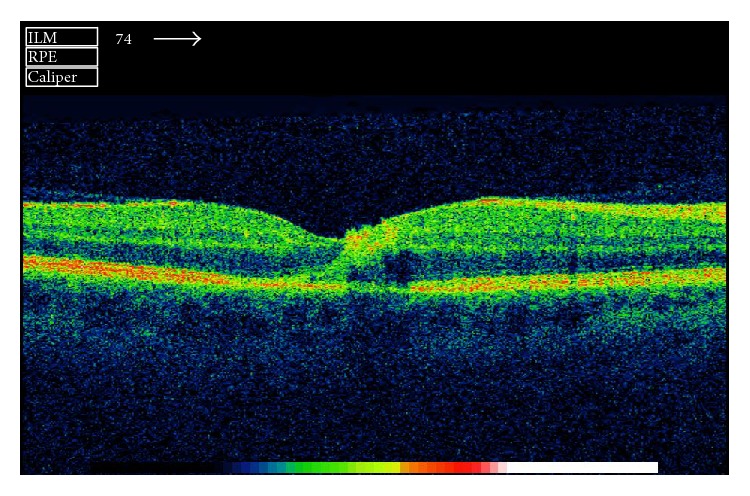
Spectral-domain optical coherence tomography scan of the right macula presenting intraretinal pseudocyst and hyperreflective deposits.

**Figure 4:**
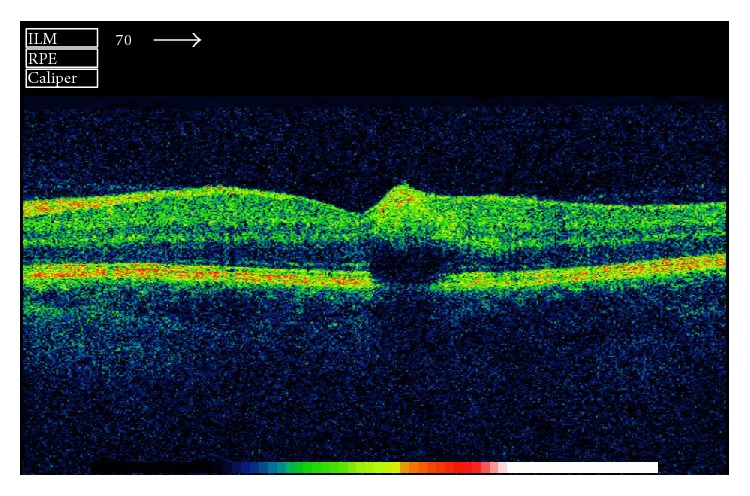
Spectral-domain optical coherence tomography scan of the left macula presenting intraretinal pseudocyst and hyperreflective deposits.

A 42-year-old man presented in the emergency outpatient clinic with reduced vision in the right eye (OD) that had developed suddenly two days before. He denied any systemic diseases though he reported intermittent muscle pain in previous weeks. The best corrected visual acuity (BCVA) (LogMAR) was 1.1 for the right eye and 0.0 for the left eye (OS). The ocular examination of the anterior segments revealed no abnormalities. Fundus examination showed white-centered hemorrhages in the macula of both eyes and near the inferior temporal vessel branches of the left eye (Figures 1 and 2). Conducted blood tests revealed notably elevated D-dimer concentration (1460.67 ng/mL; reference range: 0–500 ng/mL), slightly elevated white blood cells concentration (10.29 K/*μ*L; reference range: 4.0–10.0 K/*μ*L) with increased number of neutrophils (8.82 K/*μ*L; reference range: 2.8–6.8 K/*μ*L), high C-reactive protein concentration (29.8 mg/L; reference range: below 5 mg/L), and elevated erythrocyte sedimentation rate (26 mm/h; reference range: 2–8 mm/h). Procalcitonin concentration, platelet count, and body temperature were all within normal range. The Doppler ultrasonography showed right internal carotid artery obliteration. Consecutive fluorescent angiography did not present any leakage of fluid nor obstruction. The spectral-domain optical coherence tomography (SD-OCT) revealed intraretinal pseudocysts and hyperreflective deposits in the areas corresponding to the Roth spots (Figures 3 and 4). Two sets of blood cultures yielded* Streptococcus mitis* and intravenous cefazolin (2 g daily) was administrated immediately. During hospitalization the patient started complaining of chest and left calf pain, and due to the worsening of his general condition, he was transferred to the department of internal medicine. On the day of transfer BCVA were 1.6 OD and 0.1 OS. The computed tomography angiography examination of the thoracic aorta showed ascending aorta widening and partial spleen infarct. The following transesophageal echocardiogram revealed bicuspid aortic valve regurgitation and signs of infective endocarditis (aortic valve vegetation) with paravalvular abscess. The patient was transmitted to the cardiac surgery department where he underwent prosthetic aortic valve implantation. Three months after the surgery ophthalmologic examination showed no changes in eye fundus nor OCT abnormalities and BCVA equaled 0.3 OD and 0.0 OS. Further six weeks later the visual acuity improved to 0.0 OD as well.

## 3. Discussion

Roth spots are named after Moritz Roth (1849–1914), a Swiss physician who described them in 1872 as* retinitis septica* [2]. These white-centered hemorrhages are an ocular manifestation of systemic disorders involving impaired coagulation or capillary fragility. Although Roth spots have been mainly associated with subacute bacterial endocarditis, they can also occur in the following diseases: leukemia, anemia, carbon monoxide poisoning, hypertensive retinopathy, anoxia, preeclampsia, diabetic retinopathy, shaken baby syndrome, neonatal birth trauma, connective tissue disorders, vasculitis, or ocular decompression following trabeculectomy [1, 3, 4]. Roth spots, with associated vision deterioration, may be the first symptom of serious life-threatening general conditions that often have a subacute progression. Therefore, white-centered retinal hemorrhages should always alert the ophthalmologist to seek an underlying systemic cause. The patient should be referred to the internal medicine specialists and should undergo careful systemic evaluation. In our case, the patient's condition worsened dramatically within days though his general condition on admission was described as being very good. Apart from impaired right eye vision he did not present with any other symptoms. The reason for such a course of events was probably related to the patient's congenital cardiac abnormality, bicuspid aortic valve (BAV). Patients with BAV have been described as more susceptible to native infective endocarditis and presented with a higher incidence of aortic perivalvular abscess [5]. Being the commonest congenital cardiac abnormality in adults with an estimated prevalence of 1-2%, BAV should always be taken into account in infectious endocarditis [5, 6]. Interestingly, in our case Roth spots were located in the macula region, this in itself being a unique presentation of native subacute endocarditis. In many previously described cases Roth spots presented in more peripheral locations [7]. They were often numerous and spread out in the whole fundus [1, 3, 8] or affecting peripapillary region [4]. Roth spots may appear and disappear with great rapidity (even within half an hour). Depending on their number and location they can compromise the vision with varying degrees. Roth spots may be also accompanied by other retinal abnormalities such as preretinal or subhyaloid hemorrhages [9, 10]. The OCT findings of the Roth spots include intraretinal pseudocysts and hyperreflective deposits; the latter have been described before with coexisting retinal nerve fibre layer thickening [4]. Retinal changes usually regress when the underlying disease is treated and vision improves with time. In our case, it took the patient over four months to regain the full vision acuity. His sight was still impaired on his first check-up after the cardiac surgery, although ophthalmologic examination presented no fundus abnormalities. Though Roth spots have been described before, their bilateral macular setting remains an extremely rare condition [10, 11].

## References

[1] Ling R., James B. (1998). White-centred retinal haemorrhages (Roth spots). *Postgraduate Medical Journal*.

[2] Roth M. (1872). Uber netzhautuffecstionen bei wundfiebrin. *Deutsche Zeitschrift für Chirurgie*.

[3] Pomeranz H. D. (2002). Roth spots. *Archives of Ophthalmology*.

[4] Zehetner C., Bechrakis N. E. (2011). White centered retinal hemorrhages in vitamin B_12_ deficiency anemia. *Case Reports in Ophthalmology*.

[5] Mordi I., Tzemos N. (2012). Bicuspid aortic valve disease: a comprehensive review. *Cardiology Research and Practice*.

[6] Ward C. (2000). Clinical significance of the bicuspid aortic valve. *Heart*.

[7] Hess R. L. (2013). Roth spots in native valve endocarditis. *The Journal of the American Osteopathic Association*.

[8] Ramaswamy A., Tarabishy A., Gugliotti D., Harte B. (2011). Roth spots—more than meets the eye. *Journal of Hospital Medicine*.

[9] Vose M. J., Charles S. J. (2003). Roth's spots: an unusual presentation of HIV. *Postgraduate Medical Journal*.

[10] Priluck J. C., Chalam K. V., Grover S. (2012). Spectral-domain optical coherence tomography of Roth spots in multiple myeloma. *Eye*.

[11] Mahroo O. A., Graham E. M. (2014). Roth spots in infective endocarditis. *The New England Journal of Medicine*.

